# Molecular mechanisms underlying methotrexate-induced intestinal injury and protective strategies

**DOI:** 10.1007/s00210-024-03164-x

**Published:** 2024-06-01

**Authors:** Gaber F. Ali, Emad H. M. Hassanein, Wafaa R. Mohamed

**Affiliations:** 1 Department of Pharmacology and Toxicology, Faculty of Pharmacy, Beni-Suef University, Beni Suef, 62514 Egypt; 2 Department of Pharmacology & Toxicology, Faculty of Pharmacy, Assiut Branch, Al-Azhar University, Assiut, 71524 Egypt

**Keywords:** Intestinal toxicity, Inflammation, Methotrexate, NF-κB, Nrf2/HO-1, JAK/STAT3

## Abstract

Methotrexate (MTX) is a folic acid reductase inhibitor that manages various malignancies as well as immune-mediated inflammatory chronic diseases. Despite being frequently prescribed, MTX’s severe multiple toxicities can occasionally limit its therapeutic potential. Intestinal toxicity is a severe adverse effect associated with the administration of MTX, and patients are significantly burdened by MTX-provoked intestinal mucositis. However, the mechanism of such intestinal toxicity is not entirely understood, mechanistic studies demonstrated oxidative stress and inflammatory reactions as key factors that lead to the development of MTX-induced intestinal injury. Besides, MTX causes intestinal cells to express pro-inflammatory cytokines like interleukin-6 (IL-6) and tumor necrosis factor-alpha (TNF-α), which activate nuclear factor-kappa B (NF-κB). This is followed by the activation of the Janus kinase/signal transducer and activator of the transcription3 (JAK/STAT3) signaling pathway. Moreover, because of its dual anti-inflammatory and antioxidative properties, nuclear factor erythroid-2-related factor 2/heme oxygenase-1 (Nrf2/HO-1) has been considered a critical signaling pathway that counteracts oxidative stress in MTX-induced intestinal injury. Several agents have potential protective effects in counteracting MTX-provoked intestinal injury such as omega-3 polyunsaturated fatty acids, taurine, umbelliferone, vinpocetine, perindopril, rutin, hesperidin, lycopene, quercetin, apocynin, lactobacillus, berberine, zinc, and nifuroxazide. This review aims to summarize the potential redox molecular mechanisms of MTX-induced intestinal injury and how they can be alleviated. In conclusion, studying these molecular pathways might open the way for early alleviation of the intestinal damage and the development of various agent plans to attenuate MTX-mediated intestinal injury.

**Graphical Abstract:**

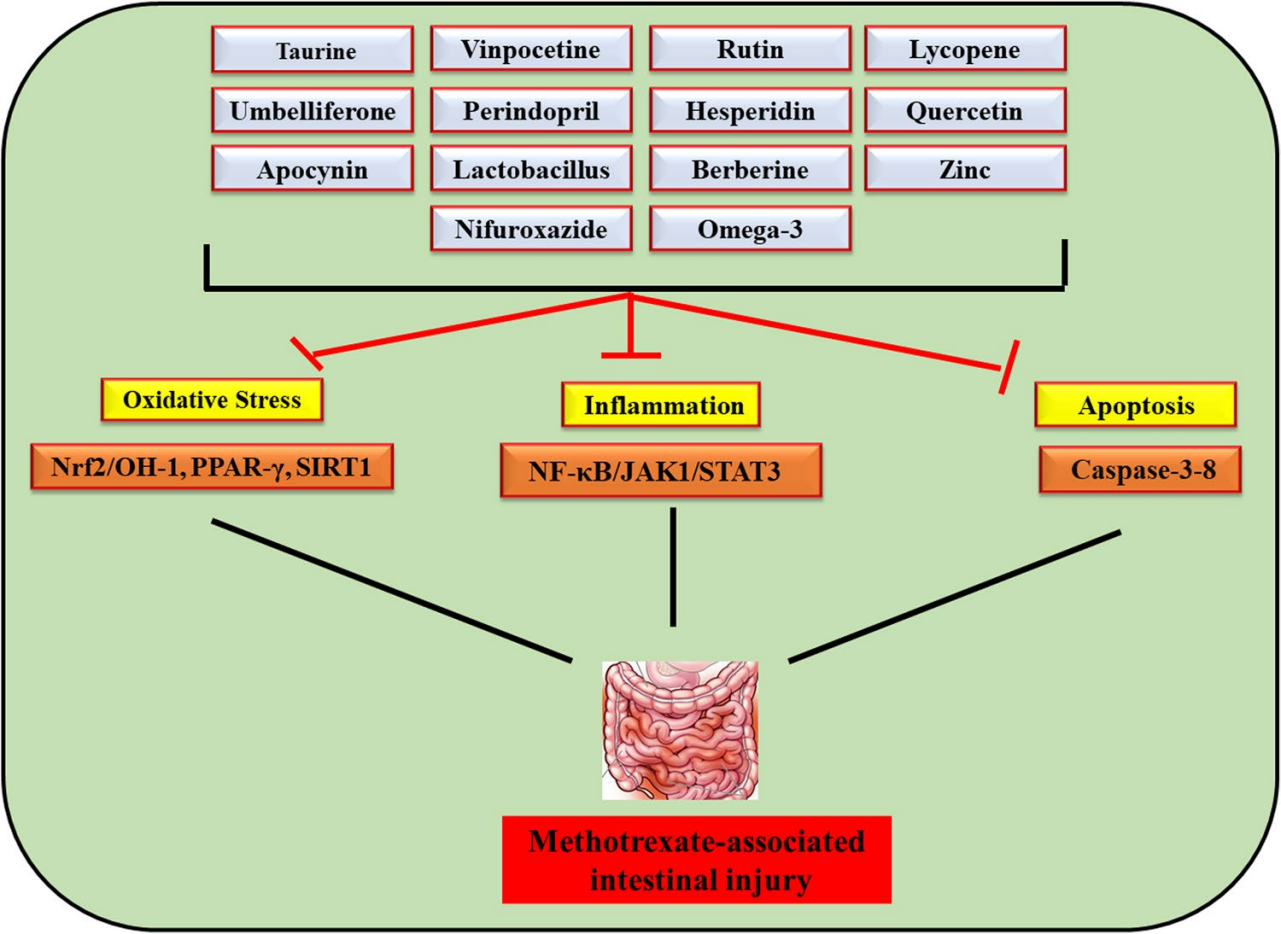

## Introduction

Intestinal mucosal injury is a common unfavorable side effect of chemotherapy, which arises from the drugs’ inability to distinguish between normal and tumor cells. The intestinal epithelial cells are frequently attacked during chemotherapy treatments due to their ability to proliferate quickly (Dahlgren et al. 2021). Up to 25–75% of cancer patients undergoing various chemotherapy treatments experience chemotherapy-induced intestinal mucosal injury which can include diarrhea, a decline in quality of life, treatment intolerance that forces discontinuation of drugs, and even mortality (Li et al. 2023). Methotrexate (MTX), a chemotherapeutic medication that has a folate antagonistic effect, is frequently used to treat multiple types of cancers, including breast cancer and lymphoma as well as immune-mediated inflammatory chronic diseases (Joerger et al. 2012). Unfortunately, MTX cytotoxicity is not limited to cancer cells but extends to affect non-cancer cells of vital organs such as intestinal mucosa (Tang et al. 2020). Gastrointestinal toxicity by MTX causes nausea, vomiting, and loss of nutrient absorption. Enteritis is distinguished histologically by crypt loss and atrophy of intestinal villi (Miyazono et al. 2004). Administration of MTX impairs mucosa barrier function, which causes bacterial translocation and inflammation. Also, its administration results in intestinal damage involving notable morphological small intestine injury and mucosal damage (Huang et al. 2020). Besides, MTX treatment causes DNA strands to break in intestinal epithelial cells that proliferate quickly (Sonis 2004) and induces oxidative stress (El-Sheikh et al. 2016; Gautam et al. 2016). More significantly, MTX may have harmful consequences by inducing a dynamic series of inflammatory events in the intestinal epithelium and submucosal tissues that are initiated by direct cellular damage (Sonis 2004; Sonis et al. 2004). Consequently, the purpose of the underlying review is to elucidate the potential molecular mechanisms of MTX-induced intestinal injury and study the protective strategies involved in the amelioration of this injury. In particular, we aimed to assess the role of inflammation and oxidative stress with a focus on the nuclear factor-kappa B (NF-κB), the Janus kinase/signal transducer and activator of the transcription3 (JAK/STAT3), nuclear factor erythroid-2-related factor 2/heme oxygenase-1 (Nrf2/HO-1), peroxisome proliferator-activated receptor-gamma (PPAR-γ), and silent information regulator-1 (SIRT1) in pathogenesis of intestinal injury induced by MTX. A deeper understanding of the molecular mechanisms involved in MTX-induced intestinal injury may help to explain a number of the drug’s toxicities and develop multiple strategies to be investigated to ameliorate the harmful adverse effects of MTX.

## Chemical properties of methotrexate

**Fig. 1:**
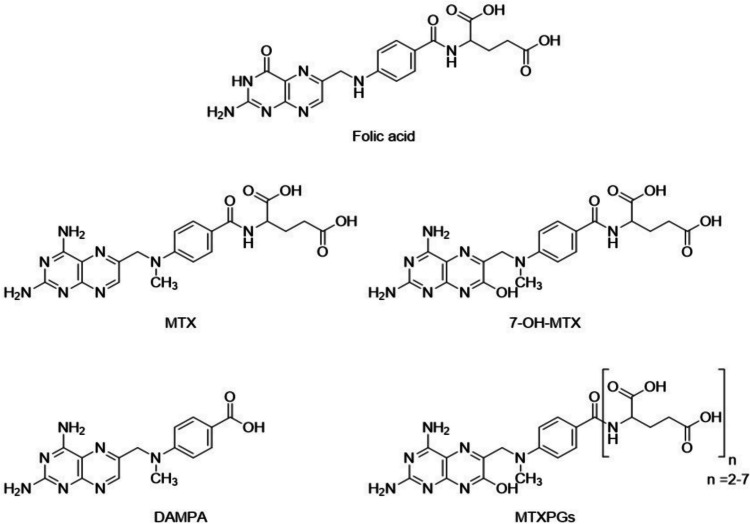
Chemical structures of folic acid, MTX, and its main metabolites

The antifolate medication MTX, also referred to 4-amino-N10-methylpteroylglutamic acid, was developed in 1940 as the first anticancer medication (Abolmaali et al. 2013). MTX and folic acid have remarkably similar structures. The structure of MTX consists of a pteridine-diamine core and a p-amino benzoyl portion connected to a glutamic acid segment containing two highly ionizable carboxylic acid groups. Since MTX must dissolve in neutral or basic solutions, its solubility is pH-dependent. S and R stereoisomers are the product of an asymmetric carbon in the molecule. The R isomer is considered an impurity, and S-MTX is regarded as the active form (Guichard et al. 2017). It is evident that the substitution of an amino function for the hydroxyl group on C2 and the methylation of N10 are the primary structural differences between MTX and the structure of naturally occurring folic acid (Rubino 2001). The structures of folic acid, MTX, and its three main metabolites are illustrated in Fig. 1.

## Indication of methotrexate

MTX is an FDA-approved drug for treating rheumatoid arthritis patients. It may also be useful in patients suffering from juvenile idiopathic arthritis (Braun 2010). MTX was used initially in rheumatoid arthritis after a double-blind, placebo-controlled clinical trial of MTX in rheumatoid arthritis patients (Weinblatt et al. 1985). Nowadays, MTX is one of the main chemotherapeutic options for treating different kinds of cancer. It is frequently used to manage several cancer types including lung cancer, lymphoma, bladder cancer, and breast cancer (Joerger et al. 2012; Khan et al. 2012) and at low doses for many autoimmune illnesses like systemic lupus erythematosus (Cipriani et al. 2014; Bedoui et al. 2019). Besides, European and American guidelines recommend the use of MTX for active Crohn’s disease and psoriasis (Gomollón et al. 2017; Coates et al. 2020; Nielsen et al. 2020). Additionally, MTX has demonstrated efficacy when paired with anti-tumor necrosis factor-alpha (TNF-α) drugs in the treatment of individuals suffering from ulcerative colitis, breast cancer, lung carcinoma, head and neck malignancies, and ovarian carcinoma (Chande et al. 2014).

## Mechanism of action of methotrexate in cancer and autoimmune diseases

MTX has a special mode of action when used in chemotherapy and immunosuppression in autoimmune conditions. In cancer, MTX acts as an antifolate antimetabolite. When MTX enters a cell through carriers referred to as human-reduced folate carriers (SLC19A1), polyglutamate synthetase attaches glutamate residues to the γ-carboxylate group of MTX, converting it into methotrexate polyglutamates (MTX-PGs). Glutamyl hydrolase, on the other hand, converts MTX-PGs back into MTX. Dihydrofolate reductase (DHFR) which catalyzes the conversion of dihydrofolate into tetrahydrofolate, the active form of folic acid, is competitively inhibited by MTX and MTX-PGs, which depletes vital tetrahydrofolate (THF) needed for cellular functions (Xu et al. 2022). DNA and RNA synthesis is inhibited by the dual inhibitory action of MTX-PGs on thymine synthase and DHFR, which results in the inhibition of purine and pyrimidine de novo synthesis (Giletti and Esperon 2018; Cheng et al. 2021; Mikhaylov et al. 2019). DHFR is a key enzyme in the process of thymidylate synthesis, catalyzing the folate reduction to THF in two steps: during the first step, folates are reduced to dihydrofolate (DHF), which are further reduced to THF (Neradil et al. 2012). The organism contains several active folate forms, including 5-methyl-THF, 10-formyl-THF, and 5,10-methylene-THF, which are donors of monocarbon units like methyl, formyl, and methylene (Assaraf 2006). The hydroxy methyltransferase enzyme, which is also involved in the conversion of L-serine to glycine, mediates the conversion of THF to 5,10-methylene-THF. 5,10-Methylene-THF is a carbon donor and coenzyme in the methylation of 2-deoxyuridine-5-monophosphate (dUMP) to 2-deoxythymidine-5-monophosphate (dTMP) which is being mediated by thymidylate synthase. Lack of THF directly affects de novo pyrimidine synthesis (Rao et al. 2003). THF is necessary for the synthesis of the nucleotides in both DNA and RNA. MTX-polyglutamate further suppresses DNA synthesis by blocking the de novo production of purine and thymidylate synthase (Singh et al. 2019). For autoimmune diseases, MTX is the recommended medication for several reasons. MTX-glu inhibits the folate pathway component thymidylate synthetase that promotes thymine nucleoside residue generation. Additionally, MTX-glu prevents the conversion of 5-aminoimidazole-4-carboxamide ribonucleotide (AICAR) to formaminoimidazole-4-carboxamide ribonucleotide (FAICAR) by inhibiting the key enzyme AICAR transformylase (ATIC) in the purines de novo synthesis pathway, leading to the accumulation of AICAR (Friedman and Cronstein 2019). The release of adenosine into extracellular space is promoted by the buildup of AICAR. Adenosine has an anti-inflammatory effect by interacting with receptors on neutrophils and monocytes (Whittle and Hughes 2004). Because of its anti-inflammatory properties, adenosine inhibits methyltransferase activity, which stops interleukin-1beta (IL-1β) from binding to its cell surface receptor, suppresses T-cell activation, down-regulates B-cells, and enhances the sensitivity of activated CD-95 T cells (Mikhaylov et al. 2019; Tukukino and Wallerstedt 2020). By blocking ATIC with MTX-glu, pro-inflammatory cytokines such as TNF-α, IL-1, and IL-6 can also be significantly decreased (Budzik et al. 2000).

## Administration of methotrexate

MTX is usually given as a single weekly dose in treating autoimmune disorders. In clinical practice, based on clinical response or intolerance, the starting dose of medication is 10 mg/week, with increases of 5 mg every 2–4 weeks, up to a maximum dose of 20–30 mg/week (Visser and van der Heijde 2009; Inoue and Yuasa 2014). MTX monotherapy has been included in recent clinical guidelines for the treatment and remission maintenance of active Crohn’s disease (Torres et al. 2020; Feuerstein et al. 2021). Previous publications demonstrated the effectiveness of intramuscular (IM) MTX at a dose of 25 mg/week for 12 months in a randomized controlled trial including thiopurine-naïve patients with Crohn’s disease (Feagan et al. 1995; Park et al. 2023a). The use of parenteral MTX has gained popularity recently and is more beneficial than taking it orally, especially when administered subcutaneously (SC). It has been demonstrated that SC MTX is more clinically effective and has better tolerance than the oral route. Currently, when oral MTX is not tolerated or shows insufficient clinical response, SC MTX treatment is advised (Visser and van der Heijde 2009; Bello et al. 2017).

## Pharmacokinetic of methotrexate

### Absorption

Following oral treatment, MTX is absorbed in the proximal jejunum by the proton-coupled folate transporter (PCFT/SLC46A1), which then transports reduced folates in addition to MTX (Desmoulin et al. 2012). A tiny amount of MTX may be converted by intestinal bacteria to 4-amino-4-deoxy-N10-methylpterroic acid (Grim et al. 2003). Although MTX has a relatively high bioavailability (64–90%), it varies significantly among patients and reaches a plateau at doses beyond 15 mg/week, suggesting intestinal transporter saturation (Hillson and Furst 1997; Hoekstra et al. 2004; Schiff et al. 2014). Studies have shown that SC MTX has a better bioavailability than oral MTX (Hoekstra et al. 2004; Bianchi et al. 2016).

MTX bioavailability varies from 30 to 90% in different people. Under fasting conditions, MTX’s Tmax was attained in 0.75–2 h, and its Cmax ranged from 0.3 to 1.6 µmol/L. Food does not considerably alter the bioavailability of MTX; however, it slightly prolongs the Tmax and decreases the Cmax. Various administration routes result in varying medication concentrations. When MTX is injected with IM or SC, the serum concentration is extremely high (Rajitha et al. 2017). The concentration of MTX in the synovium is significantly higher than that in the serum following intraarticular injection. The synovial MTX concentration is equivalent to the plasma concentration after oral or IM treatment (Rajitha et al. 2017).

The oral absorption of MTX is rapid but incomplete due to factors like receptor saturation, the inhibitory effect of food on its absorption, and rapid metabolization by gut flora (Attwa et al. 2019). The SC method of administration of MTX is becoming more popular, despite the oral route still being used the most commonly. The reason for this is that multiple trials have shown that the bioavailability of SC MTX is greater, and the bioavailability of oral MTX is somewhat variable. The bioavailability of oral MTX exhibits significant interpatient variability and a plateaued effect at doses over 15 mg/week. In contrast, the bioavailability of SC MTX is dose-dependent and linear, displaying no plateau (Schiff et al. 2014). In fact, some clinical trials have also suggested switching from the oral to the parenteral route of MTX administration (Jundt et al. 1993; Hoque et al. 2023). Studies on SC MTX have also revealed a good risk-benefit profile, indicating that the SC route may be superior to the oral route for administering higher doses of MTX because of the speed and sustainability of response (Warren et al. 2017; Dogra et al. 2022).

It is currently unclear what mechanism underlying the saturation and variability of oral bioavailability of MTX in clinical practice (Murakami and Mori 2012). Besides, the increase in oral bioavailability by administration of a higher oral dose of MTX suggests the contribution of saturated transport in oral bioavailability (VanWert and Sweet 2008). MTX is recognized as a substrate for several transporters, such as solute carrier (SLC) influx transporters and ATP-binding cassette transporters (ABC) efflux transporters. Regarding SLC transporters, reduced folate carrier (RFC), proton-coupled folate transporter (PCFT), organic anion transporter 3 (OAT3), and organic anion transporting polypeptide 1A2 (OATP1A2) all transport MTX as a substrate (Badagnani et al. 2006; Shibayama et al. 2006). Intestines exhibit PCFT and RFC, with PCFT being particularly expressed in the brush-border membranes of the proximal small intestine (Urquhart et al. 2010). Multiple strategies for optimizing MTX dosing regimens should be followed to ensure consistent drug exposure in patients. Oral MTX responsiveness can be enhanced by administering a large starting dose and rapidly titrating the medication; this approach does not seem to compromise safety or tolerability (Bello et al. 2017). Patients who are not able to tolerate MTX treatment or whose efficacy is insufficient can be “rescued” by converting to SC MTX. Beginning with SC MTX should also be taken into consideration due to its advantageous pharmacokinetic profile and absorption (Tornero Molina et al. 2021). Treatment persistence is probably going to be improved if patients are started on SC MTX or switch from oral to SC delivery (Li et al. 2021a).

### Distribution

MTX can be distributed to synovial fluid in amounts similar to those in plasma (Herman et al. 1989).

### Metabolism

One of MTX’s main metabolites, 7-hydroxymethotrexate (7-OH-MTX), is produced by the liver during the first-pass metabolism of MTX (Seideman et al. 1993).

### Excretion

Renal excretion is the main route of MTX elimination. The medication goes through active tubular secretion and reabsorption in addition to being filtered by the glomeruli. Bile excretes a tiny amount of MTX, and some enterohepatic recycling also takes place (Nuernberg et al. 1990; Seideman et al. 1993).

Renal elimination is the primary route of excretion for both MTX and its metabolites. This process involves glomerular filtration, tubular secretion, and tubular reabsorption. Tubular secretion and reabsorption have high interindividual variability, and both can be saturated which can result in nonlinear pharmacokinetics (Van Roon and Van De Laar 2010; Maksimovic et al. 2020). Between 2 and 12% of patients receiving high-dose MTX therapy may get acute kidney injury, mostly as a result of crystal nephropathy caused by MTX and its metabolite, 7-OH-MTX. Under acidic conditions, MTX and its metabolite, 7-OH-MTX, precipitate pH-dependent crystals within the tubular lumen of renal tubules. Urine alkalinization dramatically improves MTX and 7-OH-MTX solubility and excretion reducing medication toxicities (Howard et al. 2016; Reed et al. 2019). Since more than 90% of MTX is excreted by the renal tubules, any kidney problem could result in inefficient elimination of MTX. As a result, there may be a notable rise in MTX-related toxicities due to prolonged, persistent, or elevated MTX plasma levels. Renal impairment was thought to be caused by the precipitation of MTX and its metabolites in the renal tubules. Previous publications stated that renal tubular enlargement and MTX-induced kidney failure are the two reasons why MTX causes renal failure (Grönroos et al. 2006; Hamed et al. 2022). Therefore, as soon as MTX treatment starts, routine monitoring of serum creatinine and plasma MTX levels is crucial to predict the onset of renal failure. Recent studies have demonstrated the use of biomarkers for kidney impairment, including kidney injury molecule-1 (KIM-1) and cystatin C in the diagnosis of kidney impairment (Hagos and Wolff 2010; van Meer et al. 2014).

#### Factors affecting MTX pharmacokinetics

Multiple factors contribute to the variability in MTX bioavailability among patients. Age is considered a crucial factor in both pediatric and adult populations. Delayed MTX excretion increases with age as indicated by a previous study (Zang et al. 2019; Yang et al. 2024). Additionally, total protein, albumin, and globulin levels may have some influence on MTX’s clearance because of its about 50% protein binding rate (Mei et al. 2018). Urine pH has been implicated in MTX bioavailability. Both MTX and 7-OH-MTX show limited solubility in water under acidic circumstances (pH 5–7), with 7-OH-MTX having a solubility of three to five times lower than MTX (Schofield et al. 2015). Since urine pH is directly related to renal injury, in patients receiving MTX treatments, low urine pH in the early stages of treatment is a substantial independent risk factor for MTX-induced nephrotoxicity (Kawaguchi et al. 2021). MTX is mostly taken up by cells via the SLC superfamily of transporters such as SLC19A1, SLC21, SLC22, and SLC46A and can be pumped out by different ABC (Desmoulin et al. 2012). Multiple membrane-bound proteins make up the human SLC transporter family. This family influences the development of many human diseases due to the physiological and pharmacological roles of its members. For this reason, research on SLC transporters is a crucial area for the study of therapeutic medications. In addition, gene polymorphisms in SLC transporters impact drug efficacy and toxicity (Schaller and Lauschke 2019). The solute carrier family 19 member 1 (SLC19A1) is encoded by the RFC1 gene. The second exon of RFC1 gene known as rs1051266 (80 G > A) has the most prevalent single nucleotide polymorphism (SNP), which causes arginine to histidine substitution, therefore modifying the transport capacity of MTX and subsequent pharmacokinetic profile of MTX (Giletti and Esperon 2018; Xu et al. 2022).

## Adverse effects of methotrexate

Intestinal inflammation and injury are a common side effect of MTX treatment, which are caused by elevation in oxidant parameters and a decline in antioxidant status (Ozcicek et al. 2020). Additionally, various difficult toxicities associated with MTX such as testicular toxicity, which is regarded as a severe adverse effect that may result in infertility, may limit the drug’s therapeutic impact (Howard et al. 2016). Liver injury is also developed following MTX administration characterized by elevated liver function biomarkers (Bannwarth et al. 1994; Ezhilarasan 2021). As a member of category X medication, MTX is not recommended for usage during pregnancy. If this treatment is provided to a female of reproductive age, she must be aware of the possibility of teratogenesis and be instructed to use double contraception. Patients may also get mucosal ulcers when taking large doses. Other potentially severe side effects include gastrointestinal bleeding, pancreatitis, alopecia, lethargy, high body temperature, low white cell count, infections, and interstitial pneumonitis (Kremer et al. 1995; Gohar 2019; Yang et al. 2024). Generally speaking, toxicity rather than ineffectiveness is the primary reason for stopping MTX treatment (Romao et al. 2014). Periodic, meticulous, and sufficient patient monitoring appears to considerably reduce the dangers associated with the administration of MTX (Braun 2010). A better knowledge of MTX’s molecular mechanisms of action could aid in the explanation of many toxicities associated with the drug (Tian and Cronstein 2007).

## Molecular mechanisms promoting intestinal injury in methotrexate injury

**Fig. 2:**
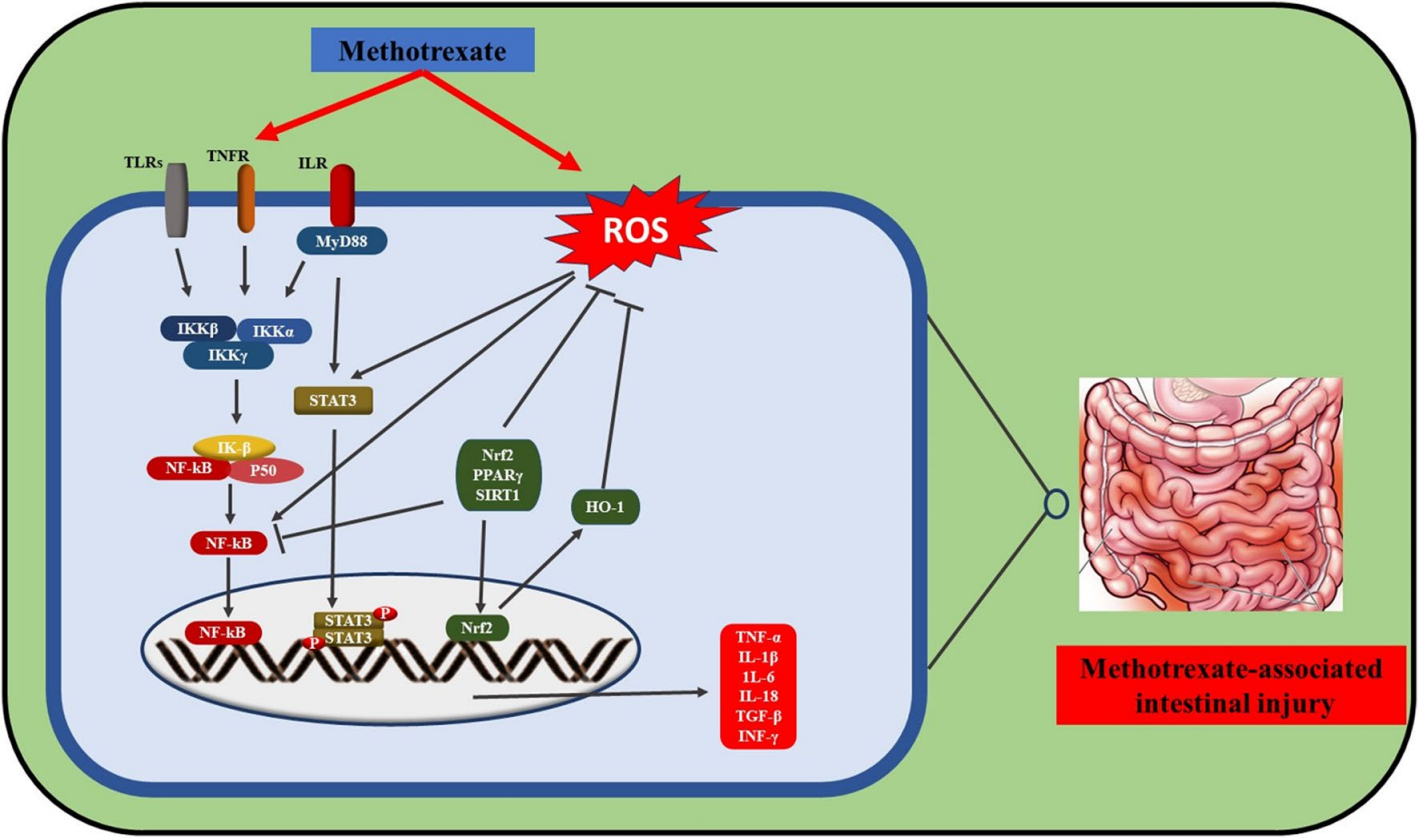
Illustration of the interplay between MTX, oxidative stress, JAK1/STAT3 pathway, and intestinal injury

The exact mechanism of intestinal toxicity caused by MTX is not fully understood. However, it was reported that MTX could cause intestinal damage via producing reactive oxygen species (ROS) and transcription factor activation as NF-ĸB (Miyazono et al. 2004; Natarajan et al. 2018). NF-ĸB regulates the production of numerous cytokines and mediates cell damage, which can be activated by ROS generation (Baeuerle and Baichwal 1997; Asehnoune et al. 2004). The production of inflammatory cytokines such as TNF- α, IL-1β, and IL-6 is provoked by ROS production (Asami and Shimizu 2021). Also, MTX administration leads to inflammatory cascades involving the activation of NF-κB, with increased expression of pro-inflammatory cytokines such as IL-6 and TNF-α, followed by activation of the JAK/ STAT3 signaling (RFd et al. 2014; Kamel et al. 2022). Upon JAK/STAT phosphorylation, it translocates to the nucleus, binds with the target gene promoter region, and provokes the transcription of genes involved in the inflammatory reactions (Rawlings et al. 2004; Xin et al. 2020) (Fig. 2).

### Role of inflammation in MTX-induced intestinal injury

Inflammation is a physiological reaction of the body towards external and internal stimuli (Pahwa et al. 2023). Leucocytes and plasma molecules are transported to tissues and infection sites via this process. Three main alterations take place during acute inflammation: increased capillary permeability, which permits larger serum molecules to enter the tissues, increased leukocyte migration into the tissue, and increased blood flow to the affected area (Al-Kofahi et al. 2017). The activation of macrophages and lymphocytes which leads to a coordinated cytokine response is a hallmark of chronic inflammation (Germolec et al. 2018). A key factor in intestinal injury pathogenesis is inflammation (Huang et al. 2020). Inflammatory cells against antigens release molecules called cytokines that regulate immunological and inflammatory responses. IL-1β and TNF-α, which are related to inflammation, are among these cytokines. Among these substances that raise inflammatory cytokines is MTX (Yucel et al. 2016). Numerous previous investigations have suggested that increased pro-inflammatory cytokine levels are crucial in the development of MTX-induced intestinal damage (Tunalı-Akbay et al. 2010; He et al. 2015; Kirbas et al. 2015).

### Involvement of nuclear factor-kappa B (NF-ĸB) in methotrexate-induced intestinal injury

NF-kB is a key regulator of inflammation that is involved in the synthesis of inflammatory mediators and activation of pro-inflammatory cytokines (Liu et al. 2017a). A cellular NF-κB regulates the expression of numerous immune system components and modulates inflammation (Li and Verma 2002). Among these are pro-inflammatory cytokines, chemokines, and inducible enzymes such as nitric oxide synthase (iNOS) and cycloxygenase-2 (COX-2). Moreover, NF-κB regulates cytokines including IL-2 and IL-12 that affect the proliferation and differentiation of lymphocytes. Consequently, deregulation of NF-κB may lead to inflammatory conditions (Yamamoto and Gaynor 2001). The cytoplasmic NF-κB complex is bound to an inhibitor of NF-kB (IκB) and exists in an inactive state. The IκB kinase (IKK) complex is activated by TNF-α and other cell stressors through a series of intermediate stages that result in IkB phosphorylation and ubiquitination, which in turn causes IkB degradation and activates NF-κB followed by nuclear translocation to bind a specific DNA sequence (As Jr 1996; Hacker and Karin 2006; Palkowitsch et al. 2008). The transcriptional activation of NF-κB regulated genes implicated in inflammation, including IL-6 and TNF-α, is the outcome of these activities (Khongthong et al. 2019). In the same context, it is believed that the production of pro-inflammatory cytokines such as TNF-α, IL-1β, and IL-6 are also thought to contribute to the intestinal damage caused by MTX. Also, TNF-α inhibitors have been shown to heal mucositis in studies on humans and animals (Logan et al. 2007; Kim et al. 2009; Kirbas et al. 2015). Earlier findings have proved that MTX administration induces a series of inflammatory cascades as evident by raised NF-κB, IL-1β, and TNF-α (Sayed et al. 2021; Abd El-Ghafar et al. 2022; Hassanein et al. 2023b). Similarly, multiple previous investigations have reported that NF-κB signaling activation is highly responsible for intestinal inflammation in MTX-induced intestinal injury (Hatada et al. 2000; Jahovic et al. 2004; Zhang et al. 2022).

### Role of JAK/STAT3/SOCS3 in MTX-induced intestinal injury

Many ligands use the intracellular signal transduction pathway known as JAK/STAT to activate target genes transcriptionally. When ligands bind to their receptors, they cause JAK phosphorylation, which then stimulates STAT phosphorylation, which subsequently controls the transcription of target genes which produce chemokines and pro-inflammatory cytokines (Villarino et al. 2017). There are four cytoplasmic protein tyrosine kinases in the JAK class: JAK1, JAK2, JAK3, and TYK2 (Laurence et al. 2012). Seven transcription factors are members of the STAT family: STAT1, STAT2, STAT3, STAT4, STAT5A, STAT5B, and STAT6 (Nicolas et al. 2013). Notably, inflammatory diseases that cause organ damage have also been associated with the JAK/STAT signaling pathway. The three primary components of the JAK/STAT signaling system are JAK, STAT, and tyrosine kinase-associated receptors (Li et al. 2015). JAK/STAT is the traditional signal pathway for multiple cytokines and growth factor production (Xin et al. 2020). When cytokines like IL-6 bind to JAK/STAT3, STAT3 becomes phosphorylated. After nuclear translocation, the phosphorylated form of STAT3 acts as a transcriptional factor that boosts the production of genes related to inflammation (Huang et al. 2016; Zhu et al. 2019). Furthermore, STATs play a crucial role in neuronal and cytokine-mediated inflammation (Kim et al. 2011). Previous studies demonstrated that MTX administration provoked JAK1 and STAT3 phosphorylation in rat models of intestinal injury and hepatotoxicity using MTX (Hassanein et al. 2021; Sherif and Al-Shaalan 2022). The regulation of the JAK/STAT system involves numerous mechanisms, one of which is the control of JAK kinase activity phosphorylation by suppressor of cytokine signaling (SOCS) proteins (Cha et al. 2015; Fouad et al. 2020; Hasan et al. 2022). The most important member of the SOCS family is SOCS3, which can block JAK/STAT3 signaling in response to mitosis, growth factors, and cytokines (Xiao et al. 2018). SOCS3 has ability to reduce JAK phosphorylation by inhibiting JAK kinase binding and competing with JAK to prevent STAT3 phosphorylation (Lin et al. 2010). Previous study showed that MTX administration showed decline in SOCS3 (de Araujo Junior et al. 2014).

### Correlation between oxidative stress and MTX-induced intestinal injury

Moderate ROS is useful for multiple physiological processes such as wound healing, tissue repair, and the elimination of invasive pathogens. On the other hand, excessive ROS production leads to oxidative stress, disturbs homeostasis, and damages human tissue. It increases cellular swelling, decreases the fluidity of the cell membrane, and damages DNA, proteins, and lipids in cells (Lushchak 2014; Zhang et al. 2016). A significant source of ROS is the gastrointestinal tract, and many gastrointestinal disorders are caused by ROS. Overexposure to oxidative stress causes intestinal inflammation and mucous epithelium apoptosis, further impairing the intestinal mucosa barrier **(**Bhattacharyya et al. 2014). When intestinal damage is triggered by MTX, oxidative stress, a consequence of an imbalance between ROS and the body’s natural antioxidant defense mechanism, is developed and plays a crucial role (Zhang et al. 2022). ROS mediates lipid peroxidation, which leads to tissue damage development after MTX administration. This degradation of cell membranes impairs normal cellular activities (Şener et al. 2006). Additionally, according to a prior study, the antioxidant glutathione (GSH) content in cells was lowered and cytosolic peroxide was elevated following MTX treatment (Kesik et al. 2009). Multiple previous studies showed that MTX treatment altered redox status in the small intestine and increased intestinal ROS biomarkers (Miyazono et al. 2004; Hassanein et al. 2021, 2022, 2023a; Sayed et al. 2022). However, the underlying mechanism by which MTX provokes tissue injury is not yet well known, and direct toxic effects of MTX are thought to be caused by excessive generation of free radicals, causing an imbalance between free radical production and antioxidant defense, which finally results in the development of oxidative stress (Drishya et al. 2022). Tissue damage developed after MTX utilization is caused by ROS which mediates destruction of lipids resulting in a breakdown of cell membrane and disturbance of physiological processes (Şener et al. 2006). Oxidative stress causes necroptosis and apoptosis in enterocytes, as well as the destruction of the intestinal structure (Zorov et al. 2006; Pi et al. 2014). In addition, cytoskeletal proteins and other cellular proteins are damaged by an overabundance of free radicals in the intestinal epithelium. Furthermore, it increases intestinal permeability, which makes it more likely for microorganisms and antigens from the luminal environment to enter the bloodstream and increase the risk of systemic reaction syndrome (Trushina and McMurray 2007). Reactive nitrogen species (RNS) and ROS have harmful cytotoxic effects on mammalian cells in living organism. The free radicals generated during oxidative stress include non-free radical species like hydrogen peroxide (H_2_O_2_) and nitrous acid, as well as different forms of activated oxygen and nitrogen such as superoxide anion (O^2•**−**^), hydroxyl, and nitric oxide (NO) radicals (Marra et al. 2011). Oxidative stress leads to lipid peroxidation, which generates a variety of oxidative compounds, including hexanal, 4-hydroxy nonanal (4-HNE), and malondialdehyde (MDA). Although 4-HNE is the most toxic byproduct of lipid peroxidation, MDA is thought to be the most mutagenic one. Additionally, oxidative stress products such as ROS covalently modify peptide bonds or amino acid side chains resulting in protein oxidation (Unsal and Belge-Kurutaş 2017). Elevated amount of ROS leads to prolonged oxidative stress and produces a potentially hazardous environment for the cells. In normal physiologic condition, there is a balance between ROS generation and antioxidative defense mechanism in the cell. A crucial role is played by endogenous antioxidant enzymes such as superoxide dismutase (SOD) and catalase (CAT) that act on O^2•**−**^ and H_2_O_2_, respectively, as well as glutathione peroxidase (Gpx) that uses GSH as co-substrate (Fu and Chung 2018).

### Nuclear factor erythroid-2-related factor 2 (Nrf2)-Kelch-like ECH-associated protein 1 (Keap1) pathway and intestinal injury by MTX

The primary regulator of cellular responses to external stressors is nuclear factor erythroid-2-related factor 2 (Nrf2) (Kobayashi et al. 2004). The nuclear factor erythroid-2-related factor 2 gene is responsible for encoding antioxidants and detoxification enzymes providing a redox sensing system (Wang et al. 2008). Kelch-like ECH-associated protein 1 (KEAP1) is a natural inhibitor of Nrf2 that negatively regulates its activity by proteasomal degradation (Singh et al. 2006). Following xenobiotic exposure, the Nrf2/Keap1 pathway is activated, releasing Nrf2 and causing it to translocate into the nucleus where it forms a heterodimer with its partner sMAF oncogene homolog. Then, it binds to the antioxidant response element (ARE) sequences regulating several targeted genes such as glutathione S-transferase (Gst) and heme oxygenase-1 (HO-1) (Taguchi et al. 2011).

The genes that encode drug-metabolizing enzymes and transporters, antioxidant enzymes, and heme and iron metabolic enzymes are among Nrf2’s target genes (Suzuki et al. 2013). The intestine and lung, two detoxifying organs or tissues that directly oppose the environment, have notably high Nrf2 expression levels (Kobayashi et al. 2004).

In a dose- and time-dependent manner, hyperactivation of Nrf2 diminished oxidative stress by ameliorating cell apoptosis and improving the redox state of the cell (Song et al. 2017). Several experimental models have been used to study Nrf2’s capacity to maintain the intestinal barrier, including *Salmonella typhi* infections (Theiss et al. 2009), colitis caused by dextran sodium sulfate (Theiss et al. 2009; Li et al. 2018), intestinal ischemic-reperfusion (Chi et al. 2015; Han et al. 2016b), intestinal mucosa damage, malfunction of the epithelial barrier brought on by traumatic brain injury (Liu et al. 2017b), and intestinal burn (Chen et al. 2016). In the same context, the previous publication reported the involvement of Nrf2 in MTX-induced intestinal injury (Katturajan and Evan Prince 2023).

By activating transcription factors like NF-κB and activator protein 1 (AP-1) and upregulating kinases like phosphatidylinositol 3-kinase (PI3K) and mitogen-activated protein kinases (MAPKs), ROS generation can cause pro-inflammatory responses (Chen and Kunsch 2004). The production of ROS has the potential to trigger immune cell activation and persistent inflammation. However, persistent inflammation can also worsen the production of ROS, creating a vicious circle (Chen and Kunsch 2004). It has been proposed that reducing ROS production can lessen inflammation. Numerous investigations have indicated that there is a strong correlation between Nrf2 and NF-κB pathways. To test it, colitis was induced in Nrf2-deficient animals by administering dextran sulfate sodium treatment. Also, mice lacking Nrf2 showed higher levels of inflammation than wild-type mice (Khor et al. 2006). In conclusion, Nrf2 activation can reduce intestinal inflammation due to direct control of inflammatory pathways by suppression of ROS production (Wen et al. 2019).

### Involvement of peroxisome proliferator-activated receptor-gamma (PPAR-γ) in MTX-induced intestinal toxicity

Peroxisome proliferator-activated receptors (PPARs) belong to the nuclear hormone receptor superfamily and are ligand-dependent transcription factors. They are essential for the metabolism of carbohydrates and lipids (Wahli et al. 1995). Vertebrates have been found to have the two PPAR isotypes: PPAR-α and PPAR-γ. The liver, kidney, testes, heart, pancreas, and smooth muscle all have high levels of PPAR-α isoform expression. For instance, adipose tissue has high levels of PPAR-γ expression, and the intestines, particularly the colon, also contain it (Auboeuf et al. 1997). In addition to being a powerful regulator of energy balance and systemic as well as cellular metabolism, PPARα also suppresses a number of inflammatory responses (Liu et al. 2018). In both white and brown adipose tissue, PPAR-γ is highly expressed and is essential for controlling lipid production, energy balance, and adipogenesis. Additionally, it is expressed in the intestines, liver, kidneys, brain, immune system, and muscles (Willson et al. 2001; Moreno et al. 2004; Grygiel-Górniak 2014). PPARs move into the nucleus after interacting with agonists, where they heterodimerize with the retinoid X receptor (RXR) to perform their function. The heterodimers stimulate the transcription of the targeted genes by binding to sequence-specific PPAR response elements (PPREs) (Berger and Moller 2002). Interestingly, it is widely known that PPAR-γ is a powerful inhibitor of ROS and inflammation (Stafeev et al. 2015). PPAR-γ carries out numerous biological functions. Its conformation changes upon activation preventing the production of pro-inflammatory mediators, which in turn prevents a range of inflammatory responses (Wang et al. 2016). The reduction of NF-kB, STAT1, and AP-1 transcriptional activity is one of the PPAR-γ anti-inflammatory actions (Ricote et al. 1999). Actually, a number of studies have also shown a metabolic advantage associated with the anti-inflammatory effects of PPAR-γ targeting (Hevener et al. 2007). It is interesting to note that PPAR-γ is a significant nuclear receptor whose beneficial antioxidant and anti-inflammatory properties have led to the investigation of a variety of disorders (Korbecki et al. 2019). Previous literature has indicated that the expression of the antioxidant defense can be induced by activated PPAR-γ (Girnun et al. 2002; Chung et al. 2009) while inhibiting inflammatory cytokine productions (Vandewalle et al. 2008). Previous studies showed that a decreased level of PPAR-γ is associated with oxidative stress development in a rat model of duodenal injury induced by MTX (Sayed et al. 2022; Mansoury et al. 2023).

### Role of silent information regulator-1 (SIRT1) in MTX-induced intestinal toxicity

Among the histone deacetylases which are referred to as sirtuin1, is the silent information regulator-1 (SIRT1) protein. Significantly, SIRT1 is critical for controlling oxidative stress and mitochondrial metabolism. By controlling antioxidant genes through the FoxO3a/proliferator-activated receptor-gamma coactivator 1 alpha (PGC-1α) complex, SIRT1 prevents the generation of superoxide (Wang et al. 2020c). Additionally, recent studies suggested that SIRT1 is an essential regulator of intestinal barrier function (Tanno et al. 2007; Tao et al. 2010). Moreover, SIRT1 activation significantly ameliorated colitis induced by dextran sulfate sodium in mice (Kwon et al. 2017). It has been demonstrated to block the NF-κB signaling suppressing the inflammatory response. According to recent reports, SIRT1 contributes to cellular lifespan extension, resistance to oxidative stress, and repair of DNA damage (Elshazly et al. 2020; Gao et al. 2021). Mice with intestinal deletion of SIRT1 have been shown to exhibit microbiota dysbiosis and aberrant activation of the inflammatory response (Wellman et al. 2017). Previous publications have demonstrated that oxidative stress significantly decreased SIRT1 activity in previously experimental models. (DiNicolantonio et al. 2022). High ROS can suppress SIRT1 function by causing oxidative changes in its cysteine residues (Salminen et al. 2013). In addition, SIRT1 induces the inhibition of NF-κB and additional pro-inflammatory mediators (Shao et al. 2014). Previous publications have demonstrated the involvement of SIRT1 in MTX-provoked intestinal injury (Sayed et al. 2022; Katturajan and Evan Prince 2023; Abd-Alhameed et al. 2024).

## Therapeutic protection against methotrexate-induced intestinal injury

### Omega-3 polyunsaturated fatty acids

Over the past decades, polyunsaturated fatty acids (PUFAs) have become a topic of interest for the public and scientific community due to their involvement in numerous metabolic and physiological conditions (Palmquist 2009). Fatty fish and seafood are the main sources of them (Han et al. 2016a). Interestingly, omega-3 FAs anti-inflammatory and antioxidant properties proved their effectiveness in both preventing and treating a wide range of illnesses (Swanson et al. 2012; Scorletti and Byrne 2013; Firat et al. 2017; Oscarsson and Hurt-Camejo 2017; Karageorgou et al. 2023). Additionally, omega-3 PUFAs exhibited potential protective effect against MTX-induced apoptosis in spleen (Elsayed et al. 2021) and intestinal mucosa (Koppelmann et al. 2021) as well as acute kidney injury induced by lipopolysaccharide (Li et al. 2021b). Interestingly, omega-3 PUFAs were evaluated in rat models of intestinal damage by MTX, and this study demonstrated that omega-3 PUFAs exhibited the ability to prevent intestinal damage and stimulate intestinal recovery. Besides, MTX + omega-3 PUFA-treated rats showed a significant decrease in enterocyte apoptosis together with reduced numbers of macrophages in conjunction with lower levels of COX-2, TNF-α, and NF-κB in the mucosa of treated rats (Koppelmann et al. 2021). This study found that omega-3 PUFAs may be used as a novel therapy for attenuating MTX-induced intestinal injury through its antioxidant, anti-inflammatory, and antiapoptotic effect.

### Taurine

Taurine, also known as 2-aminoethanesulfonic acid, is a common organic substance found in animal tissues. It accounts for 0.1% of the human body weight and is primarily found in the large intestine as well as the main component of bile (Ronalds 2019). Taurine plays a vital role in many processes, including control of osmotic pressure, the stabilization of membranes, reproduction, inflammation, and the regulation of heart muscle. Numerous studies have proved that taurine is a promising agent due to its ability to overcome oxidative stress and inflammation. It can be used to protect against a wide range of conditions in several organ systems, including the skeletal, muscular, cardiovascular, respiratory, and endocrine systems (Ahmed 2023). Taurine plays a crucial role in protecting against nervous system diseases including Parkinson’s and Alzheimer’s (Jakaria et al. 2019). Molecular studies have indicated that it may act as a neuroprotectant against stroke. In a diabetic mouse model, it reduced oxidative stress-induced neuropathy by triggering antioxidative defense signals (Agca et al. 2014). Obviously, previous publications demonstrated the protective effect of taurine against MTX-induced intestinal injury through a variety of mechanisms following careful examination. The effective modulation of cytoglobin and Keap1/Nrf2/HO-1 signals mediated its potent antioxidant effects. The inhibition of the NF-κB/iNOS signal suggests its anti-inflammatory effects. Intestinal proliferating cell nuclear antigen (PCNA) and caspase-3 suppression mediate antiapoptotic and antiproliferative effects (Hassanein et al. 2022). This study explained that taurine may be used as a promising therapy in mitigating intestinal damage provoked by MTX through the regulation of oxidative stress, inflammation, apoptosis, and proliferation.

### Umbelliferone

A naturally occurring member of the coumarin family, umbelliferone (UMB) or 7-hydroxycoumarin, is present in a wide variety of plants, including garden angelica, coriander, and carrots (Mazimba 2017). Numerous investigations have determined that UMB possesses biological properties, including anti-inflammatory (Navarro-García et al. 2011), antioxidant (Hoult and Payá 1996; Cruz et al. 2020), and anticancer (Lopez-Gonzalez et al. 2004) effects. In testicular dysfunction in diabetic heavy metal-treated mice (Allam et al. 2022; Alotaibi et al. 2023), liver injury (Shalkami et al. 2021), kidney injury (Sami et al. 2022), and liver fibrosis (Park et al. 2023b), UMB showed potent antioxidant and anti-inflammatory properties as well as decreased cell damage. According to a study reported previously (Jagadeesh et al. 2016), UMB improved heart function and reduced oxidative stress and infarct size in rats. Previous study proved the promising protective effect of UMB against MTX-induced intestinal damage by markedly improved oxidant/antioxidant status, as shown by the parallel decrease in MDA contents and the elevation of Nrf2, SOD, HO-1, and GSH levels. Additionally, it reduced the number of inflammatory cascades by inhibiting STAT3, NF-κB, IL-6, and TNF-α levels. Furthermore, the expression of Wnt and β-catenin was dramatically increased by UMB (Hassanein et al. 2023a). According to these results, UMB might be applied as a possible adjuvant treatment in MTX chemotherapy regimens to overcome intestinal injury caused by MTX through the regulation of oxidative stress and inflammatory cascades.

### Vinpocetine

Ethyl apovincaminate, also known as vinpocetine, is a nootropic substance that has been intended to manage neurological illnesses related to cerebrovascular diseases. It is a synthetic derivative of the alkaloid vincamine, which is taken from the leaves of the periwinkle plant (Mohammed et al. 2023). Moreover, vinpocetine has a strong antioxidant impact by scavenging free radicals and a strong anti-inflammatory effect by directly inhibiting IKK (Abdel-Salam et al. 2016; Nadeem et al. 2018; Zhang et al. 2018). Additionally, previous study has demonstrated the increase in cerebrovascular flow by vinpocetine in individuals with cerebrovascular illness (Patyar et al. 2011). Vinpocetine’s anti-neuroinflammatory and antioxidant pathways have been suggested to be involved in its neuroprotective impact on rotenone-induced Parkinson’s disease (Ishola et al. 2023). Vinpocetine has been studied in a rat model of duodenal injury by MTX, and this study showed that the injection of vinpocetine retained the normal histology of the crypt and villous while attenuating the dramatic histological alterations caused by MTX. Through the upregulation of intestinal Nrf2 and HO-1 expression, vinpocetine dramatically reduced oxidative stress damage. By lowering IL-1β and TNF-α levels and downregulating the expressions of NF-κB, interferon regulatory factor3 (IRF3), p-JAK-1/p-STAT-3, and vinpocetine reduced inflammation. Moreover, vinpocetine efficiently inhibited caspase-8, RIPK1, RIPK3, and MLKL to counteract intestinal necroptosis (Tashkandi et al. 2023). Due to these favorable effects, vinpocetine can be used as a complementary therapy with MTX to counteract apoptosis, inflammation, and oxidative stress by MTX.

### Perindopril

Through processes involving angiotensin II, perindopril (PER), a typical angiotensin-converting enzyme inhibitor (ACEI), has been shown to be useful in a number of cardiovascular disorders (Ancion et al. 2019). PER has also been demonstrated to have antiapoptotic, anti-inflammatory, and antioxidant properties (Varin et al. 2000, Kobara et al. 2003). Earlier study has shown that PER has a potent antioxidant and anti-inflammatory activity which be helpful in treating acute kidney injury associated with sepsis (Ali et al. 2016; Kostakoglu et al. 2020). Previous research has also demonstrated that PER can reduce drug-induced kidney damage due to its antioxidant and anti-inflammatory properties (Tang et al. 2008; Shalkami et al. 2018). Preliminary investigation also showed gastroprotective effect of perindopril through counteracting oxidative stress and inflammation induced by indomethacin in a rat model of gastric injury (Mohamed et al. 2022). Previous publication has demonstrated the potential protective effect of perindopril on intestinal injury induced by MTX. This study showed that perindopril preserved the goblet cells in the villi/crypts and reduced the histological abnormalities, indicating that the intestinal injury had been attenuated. Additionally, PER reduced intestinal MDA and increased SOD activity and GSH content along with PPAR-γ and SIRT1 cytoprotective signals to attenuate the pro-oxidant processes. These favorable effects were also associated with upregulating angiotensin (1–7) and anti-inflammatory cytokine IL-10 while downregulating the production of pro-inflammatory cytokines IL-6, IL-1β, and TNF-α. Besides, in rats with inflamed intestines, PER downregulated the toll-like receptor 4 (TLR4), NF-κB, and c-Fos/c-Jun pathways at the molecular level (Sayed et al. 2022). In conclusion, PER significantly reduced MTX-induced intestinal damage by inhibiting inflammatory pathways and increasing the antioxidant cytoprotective signals.

### Rutin

Rutin is one of the main flavonoid glycosides present in fruits and fruit peels, mainly in citrus fruits such as lemons and oranges (Nafees et al. 2015; Çelik et al. 2020). It possesses several pharmacological activities, including the ability to effectively scavenge superoxide radicals and act as an immunomodulator, anti-inflammatory, antioxidant, antihypertensive, and anti-carcinogenic (Nafees et al. 2015; Caglayan et al. 2019; Kandemir et al. 2020). The main pharmacological effects and underlying mechanism of action of rutin contribute to its antioxidant capacity through the Nrf2/ARE and anti-inflammatory properties due to NF-κB, COX-2, IL-6, and TNF-α suppression. It inhibits caspase-3 and enhances B-cell lymphoma 2 (Bcl-2) suggesting its antiapoptotic effect (Janbaz et al. 2002; Nafees et al. 2015). It has been shown that rutin can ameliorate liver and/or kidney injury induced by different agents such as lead acetate (Ansar et al. 2016), acrylamide (Ahmed and Ibrahim Laila 2018), and carbon tetrachloride (Hafez et al. 2015). According to a previous investigation, rutin protects the kidneys in diabetic nephropathy (Kamalakkannan and Prince 2006) and ischemic/reperfusion renal damage (Korkmaz and Kolankaya 2013). Rutin has been shown to inhibit renal apoptosis and inflammation caused by cisplatin by lowering the expression of caspase-3, as well as TNF-α,and NF-κB (Tambağ et al. 2021). Rutin has been evaluated in a previous study of intestinal toxicity induced by MTX, and this study showed its ability to attenuate intestinal oxidative stress changes by lowering intestinal MDA and boosting GSH content and SOD activity. Moreover, administration of rutin attenuated MTX-induced intestinal inflammation, as proved by decreased IL-2 and increased IL-4 and IL-10. Additionally, rutin was found to inhibit the enzymatic activity of COX and lipoxygenase.

It can be concluded that rutin, in a dose-dependent manner, has significant physiological, immunological, and biochemical protection against MTX-induced intestinal injury (Gautam et al. 2016). The immunoregulatory and free radical scavenging potential activity could be thought of as the explanations for rutin’s activities.

### Hesperidin

Flavonoids, which are easily derived from various vegetables and fruits and possess anti-inflammatory and antiapoptotic effects in addition to their anti-autophagic properties, have gained more attention in recent times. Hesperidin (HES), a flavanone group member, is one of these compounds (Semis et al. 2021). Citrus fruits, including lemon, orange, and grapefruit, are a good source of this natural antioxidant compound (Yurtal et al. 2020; Patel and Shah 2021). HES exhibited antiapoptotic, anti-inflammatory, vasoprotective, and anti-carcinogenic properties together with its antioxidant activity, with no known adverse effects (Çetin et al. 2016; Sheikhbahaei et al. 2016; El et al. 2017). According to reports, HES scavenges ROS, chelates metal ions, and guards against lipid peroxidation to avoid oxidative damage and cell death (Polat et al. 2016; Iskender et al. 2017). Previous studies have shown the protective effect of HES against MTX-induced hepatotoxicity (Abdelaziz et al. 2020), experimental ischemia/reperfusion testicular injury in rats (Celik et al. 2016), nephrotoxicity and hepatotoxicity induced by sodium arsenite (Turk et al. 2019), paclitaxel-induced peripheral neuropathy in rats (Semis et al. 2021), and renal ischemia-reperfusion injury in rats (Meng et al. 2020). A potential experimental investigation has assessed the protective effect of HES against intestinal damage provoked by MTX using histopathological and immunohistochemical techniques. Pretreatment with HES attenuated intestinal injuries evidenced by enhancing intestinal scoring damage and crypt injury. Additionally, administration of HES counteracted intestinal oxidative stress changes by lowering intestinal myeloperoxidase concentration. Moreover, treatment with HES attenuated MTX-induced intestinal inflammation, as proved by inhibiting INOS and IL-8 level immunostaining (Acipayam et al. 2013). In conclusion, HES significantly showed notable amelioration of intestinal damage induced by MTX through its powerful antioxidant and anti-inflammatory effect.

### Lycopene

Tomatoes and other red fruits have a high concentration of the red pigment lycopene. Many double bonds in lycopene’s chemical structure play a significant part in scavenging ROS (Abdel-Daim et al. 2019; Ibrahim et al. 2022). Because of its 11 conjugated double bonds, lycopene exhibited the highest antioxidant activity among carotenoids and phytochemicals (Saini et al. 2020). Lycopene has numerous pharmacological properties including potent and effective anti-inflammatory, immunostimulant, antibacterial, and anti-mutagenic properties (Müller et al. 2011). In addition, lycopene exhibits chemo-preventive properties against some types of cancer (Huang and Hu 2011). It was found to have a potent free radical scavenging effect during severe stressful conditions. Eating tomatoes or tomato-derived products is frequently associated with lower levels of oxidative damage to proteins, lipids, and DNA due to higher amounts of circulating lycopene (Palabiyik et al. 2013). Furthermore, it is well known that dietary lycopene supplementation shields the animal intestine’s structure and tissue from harmful events when it comes into touch with pathogens, poisons, or any other foreign antigen (Sarker et al. 2021). Previous investigations showed that lycopene administration exhibited significant protection against intestinal injury provoked by radiation in rats (Anwar et al. 2013). Due to its strong scavenging activity of free radicals, lycopene has a powerful ability to protect the kidney, liver, and nervous system from oxidative stress (Zhang et al. 2020). Lycopene has recently shown obvious neuroprotective effects in several conditions involving neuroinflammatory conditions. These advantageous outcomes were due to NF-κB suppression, the maintenance of mitochondrial integrity, and the reduction of apoptosis in neurons (El Morsy and Ahmed 2020).

Lycopene was studied in a previous study investigating its protective effect against MTX-provoked intestinal injury, and this study showed that when lycopene was administered to the MTX group, the small intestinal histological damage showed a considerable recovery. Additionally, the intestinal levels of IL-1β, total oxidant status (TOS), and oxidative stress index (OSI) were dramatically reduced (Yucel et al. 2016). Because lycopene counteracts oxidative stress, inflammation, and NF-κB activation created by MTX, it may be an excellent adjuvant therapy taken with MTX.

### Quercetin

Of all the flavonoids, quercetin (3, 39, 49, 5, 7-pentahydroxyflavone) is the most widely distributed. Apples, potatoes, soybeans, and other fruits and vegetables are rich sources of quercetin (Mao et al. 2018). Strong cytoprotective and antioxidative properties of quercetin help in inhibiting endothelial cell apoptosis induced by oxidants (Choi et al. 2003). Besides, by stopping lipid peroxidation and scavenging oxygen free radicals, quercetin inhibits oxidative damage and cell death (Wang et al. 2020a). Quercetin is a powerful antioxidant that protects against ROS and has been shown to have remarkable protective benefits against diabetes, cardiovascular disease, inflammation, cancer, and damage to nerves and the eyes (Carrillo-Martinez et al. 2024). Previous publication has investigated the ability of quercetin to attenuate small intestine damage and improve intestinal recovery in MTX-induced intestinal mucositis in rats. When quercetin was administered to rats treated with MTX, a significant reduction in the intestinal injury score, a significant increase in the intestinal and mucosal weight in the ileum and jejunum, and an increase in the ileum’s protein content and villus height were observed in comparison to the MTX group (Sukhotnik et al. 2018).

### Apocynin

Apocynin (APO) (4-hydroxy-3-methoxyacetophenone) is a natural organic methoxy-substituted catechol compound that acts as an antioxidant. It is separated from *Apocynum cannabinum* root and *Picrorhiza kurroa Royle ex Benth*, a traditional medicinal plant, that belongs to the *Scrophulariaceae* family (Nwokocha et al. 2020). The primary enzyme that produces ROS is nicotinamide adenine dinucleotide phosphate oxidase (NOX), and blocking this enzyme offers a significant therapeutic target for the management of numerous illnesses (Auten et al. 2009; Datta et al. 2015). APO efficiently blocks NOX in activated leukocytes to stop ROS generation (Stefanska and Pawliczak 2008). APO has been shown in numerous animal and cell culture experiments to decrease neutrophil chemotaxis and neutrophil oxidative burst, which in turn reduces neutrophil-mediated cell damage (Stolk et al. 1994; Impellizzeri et al. 2011). According to previous investigations, APO ameliorated lung damage by reducing lipid peroxidation, inhibiting NOX expression and activity, blocking the NF-κB pathway, and suppressing the transcription of pro-inflammatory cytokines in lung tissue (Kim et al. 2012; Choi et al. 2017). A previous study investigated the protective role of APO against MTX-induced intestinal mucositis. The conserved histology of goblet cells (villi and crypts) indicates that APO preserved the histological structure of the duodenal mucosa. Besides, APO reduced intestinal oxidative stress by decreasing intestinal MDA and increasing SOD activity and GSH content. APO exhibited powerful anti-inflammatory by inhibiting the production of NF-κB mRNA and decreasing pro-inflammatory cytokine levels together with upregulating anti-inflammatory PPAR-γ proteins. Furthermore, the intestinal mucosa of rats that received APO + MTX revealed weak positive immunological staining for cleaved caspase-3 (Mansoury et al. 2023). The findings suggest that APO may be used as a potential treatment drug to stop mucositis caused by MTX due to counteracting oxidative stress, inflammatory, and apoptotic pathways.

### *Lactobacillus*

A genus of gram-positive anaerobic or microaerophilic, rod-shaped, non-spore-forming bacteria is called *Lactobacillus*. They are an important component of the microbiota in humans and can be found in the urinary, genial, and digestive systems (Wanchao et al. 2018). Moreover, *Lactobacillus* scavenges ROS to prevent oxidative damage development (Kong et al. 2020). Besides *Lactobacillus* administration diminishes ROS such as hydroxyl radicals, superoxide anions, and peroxide radicals (Wang et al. 2017). Research conducted on pigs has demonstrated that a diet containing *Lactobacillus* raises muscle SOD, CAT, and serum SOD (Wang et al. 2009). *Lactobacillus* has been shown to have a variety of anti-carcinogenic effects due to its ability to antagonize proliferation, apoptosis, and oxidative stress (Nowak et al. 2019). *Lactobacillus* shields the intestines against several intestinal damage models (Jian et al. 2022; Hassanein et al. 2023a). In the same context, *Lactobacillus* showed a protective effect against a rat model of 5-aminosalicylic-induced ulcerative colitis through the Nrf2/Ho-1 pathway and gut microbiota modulation (El-Baz et al. 2020). A previous publication has proved the intestinal protective effect of *Lactobacillus* acidophilus LB strain (LB) against MTX-induced intestinal injury. Pretreatment with *Lactobacillus* attenuated intestinal injury as evidenced by improvement of intestinal histopathology. *Lactobacillus* treatment attenuated intestinal oxidative stress changes by lowering intestinal MDA and boosting GSH content, SOD3 activity, Nrf2, and OH-1. Moreover, administration of *Lactobacillus* attenuated MTX-induced intestinal inflammation, as proved by inhibiting TNF-α, IL-6, STAT3, and NF-κB (Hassanein et al. 2023a). In conclusion, by restoring the oxidant/antioxidant balance and reducing the inflammatory burden, a pretreatment regimen with *lactobacillus* may be a potential therapeutic approach for attenuating intestinal injury caused by MTX.

### Berberine and zinc

Berberine, a well-known natural isoquinoline alkaloid, is found in a variety of plants, such as Coptis and Berberis (Cicero and Baggioni 2016). It is interesting to note that traditional medicine uses berberine to treat intestinal disorders and diarrhea (Tan et al. 2019). It also has anti-inflammatory, antioxidant, and anticancer properties (Tan et al. 2011; Deng et al. 2019; Hassanein et al. 2019). Significantly, it was documented that berberine prevented ulcerative colitis provoked by dextran sulfate sodium (Zhu et al. 2019). Obviously, berberine has been shown to have a potent anti-inflammatory impact in cases of severe abdominal infection and to mitigate intestinal mucosa injury due to stressful conditions (Wang et al. 2020b). Zinc (Zn) is a vital trace element that has anti-inflammatory and antioxidant effects and is involved in numerous biological processes (Haase et al. 2008; Marreiro et al. 2017). Previous publications have shown that Zn attains a beneficial protective effect on intestinal injury conditions. They found that Zn in the intestinal lumen can decrease sensitivity to damage and increase mucosal repair and restoration. Besides, they reported that Zn supplementation improved the gut’s ability to heal from MTX-induced injury (Tran et al. 2003; Musa et al. 2015). A previous study showed that berberine and/or Zn exhibited a notable protective effect against MTX-induced intestinal toxicity. Berberine and/or Zn ameliorated oxidative stress and enhanced changes in SIRT1, Nrf2, forkhead box-O3 (FOXO-3), JAK1, and STAT3 (Hassanein et al. 2021). Berberine and Zn may be possible medications for the intestinal damage management produced by MTX by altering the signaling pathways involved in oxidative stress and inflammation.

### Nifuroxazide

Nifuroxazide (NIF) is a highly safe oral antidiarrheal antibiotic that has been approved for the treatment of several gastrointestinal infections (Hassan et al. 2021). NIF, an antidiarrheal antibiotic, demonstrated efficacious suppression of STAT3 activation in cell lines of colorectal cancer, multiple myeloma, colon ulcer, and diabetic kidney tissues (Althagafy et al. 2023). Previous research demonstrated that NIF inhibited NF-κB signaling in liver failure induced by thioacetamide in rats (Khodir and Said 2020) and acetic acid-induced ulcerative colitis (Yousra et al. 2020). Remarkably, NIF also improved rat diabetic nephropathy by inhibiting pro-inflammatory cytokine production, oxidative stress, and NF-κB activation in diabetic kidneys (Elsherbiny et al. 2018). Regarding the impact of NIF on MTX-induced intestinal injury, NIF exhibited potent antioxidant benefits against MTX-provoked intestinal injury by controlling PPAR-γ, SIRT1, and Nrf2 redox-sensitive signals expression (Abd-Alhameed et al. 2024). In addition, intestinal inflammation was reduced by NIF due to suppression of NF-κB protein expression, downregulation of JAK1/STAT3 phosphorylation, and reducing the release of pro-inflammatory cytokines such as IL-6, IL-1β, and TNF-α. Furthermore, the histological analysis showed that NIF decreased the invasion of inflammatory cells, maintained the goblet cells, and improved the pathological alterations in the intestines. Consequently, NIF may be a good option as MTX adjuvant therapy via counteracting oxidative stress, inflammation, and NF-κB activation caused by MTX.

## Conclusions and perspectives

**Fig. 3:**
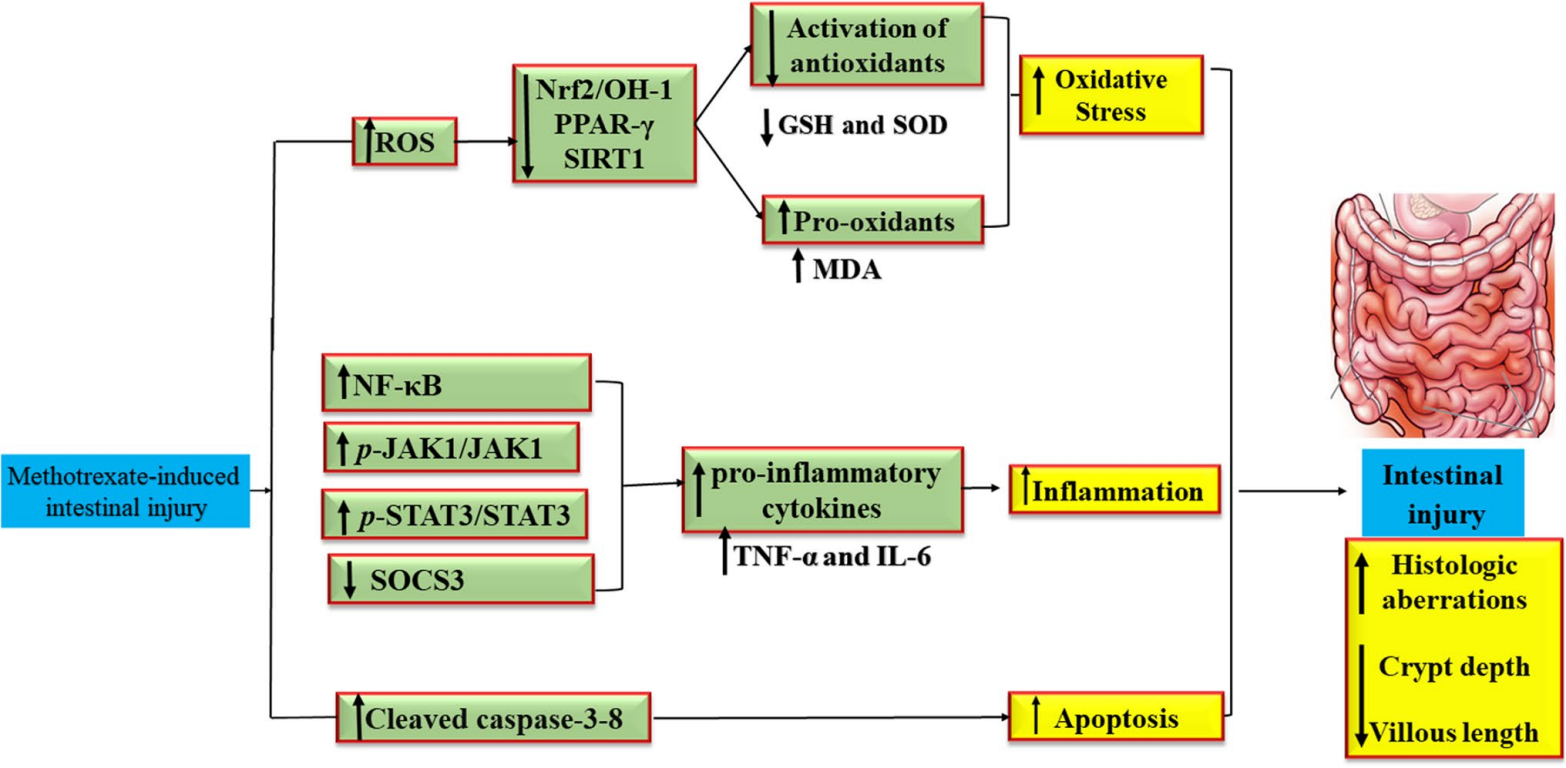
Detailed mechanism of signaling molecular pathways involved in MTX-induced intestinal injury. MTX-induced intestinal injury by increasing oxidative stress characterized by decreasing the activation of antioxidants GSH and SOD and increasing pro-oxidant MDA mediated by the downregulation of Nrf2/HO-1, PPAR-γ, and SIRT1. Also, MTX administration enhanced inflammation characterized by increasing pro-inflammatory cytokines TNF-α and IL-6 mediated by the upregulation of NF-κB and JAK/STAT3 phosphorylation and decreasing SOCS3. Furthermore, it increased apoptosis characterized by an elevation of cleaved caspase-3 and caspase-8. Abbreviations: GSH, glutathione; IL-6, interleukin-6; JAK/STAT3, Janus kinase/signal transducer and activator of transcription3; MDA, malondialdehyde; MTX, methotrexate; Nrf2/HO-1, nuclear factor erythroid-2-related factor 2/heme oxygenase-1; NF-κB, nuclear factor-kappa B; PPAR-γ, peroxisome proliferator-activated receptor-gamma; SOD, superoxide dismutase; SOCS3, suppressor of cytokine signaling3; SIRT1, silent information regulator-1; TNF-α, tumor necrosis factor-alpha

MTX is a well-known cytotoxic medication that is frequently used for managing autoimmune diseases and malignancies. Using MTX may be associated with intestinal mucous membrane damage as well as mucositis, which impairs the ability of patients to tolerate treatment and disturbs their nutritional status. Due to limited treatment options and severe adverse effects associated with MTX, multiple approaches strongly need to be investigated to counteract these severe adverse effects. By inducing lipid peroxidation and excessive ROS, MTX induces a series of oxidative stress of the intestinal mucosal membrane. Besides, it triggers the release of pro-inflammatory cytokines like NF-kB, IL-6, IL-1β, and TNF-α as well as the activation of many pro-inflammatory signaling pathways via ROS-driven mechanisms. Various molecular pathways are also involved in MTX-induced intestinal injury including JAK/STAT3/SOCS3, Nrf2/OH-1, PPAR-γ, and SIRT1. Thus, individuals with rheumatoid arthritis, psoriasis, and cancer who take MTX need to be monitored for intestinal injury. Multiple compounds such as omega-3 polyunsaturated fatty acids, taurine, umbelliferone, vinpocetine, perindopril, rutin, hesperidin, lycopene, quercetin, apocynin, lactobacillus, berberine, zinc, and nifuroxazide have been studied in previous publications and were reported to have potential protective effects in ameliorating MTX-provoked intestinal injury. As a result, novel treatment approaches for managing and alleviating MTX-induced intestinal injury will soon be required. Possible molecular mechanisms involved in MTX-induced intestinal injury and mechanisms of actions of protective agents are graphically illustrated in Fig. 3.

Hence, more investigations are needed to assess other signaling molecular pathways involved in MTX-induced intestinal injury to develop novel strategies for amelioration of intestinal injury induced by MTX. Also, further clinical studies should be done to emphasize the potential uses of previously mentioned protective agents as a potential supplementary therapy for preventing MTX-provoked intestinal injury.

## Data Availability

No datasets were generated or analysed during the current study.
